# Reconstruction of an Amputated Thumb by Great Toe Harvested from a Simultaneously Amputated Lower Limb: Spare Part Surgery Concept Revisited

**DOI:** 10.1055/s-0045-1810110

**Published:** 2025-08-06

**Authors:** S Raja Sabapathy, Hari Venkatramani, Madhu Periasamy, Aditya Narasimhan, Monusha Mohan, Vamseedharan Muthukumar

**Affiliations:** 1 Department of Plastic, Hand and Reconstructive Microsurgery, Ganga Hospital, Coimbatore, Tamil Nadu, India

**Keywords:** toe transfer, thumb reconstruction, spare part surgery

## Abstract

Utilization of tissues from the amputated part to cover critical structures or enhance function in another area is a valuable technique in reconstructive surgery. For this “spare part surgery” to occur high level of awareness is to be present among surgeons who perform the primary procedure. We are presenting a case wherein the great toe was harvested for thumb reconstruction in a patient who had a nonsalvageable lower limb injury with a crush injury of the hand with amputation of multiple fingers including the thumb. The patient on follow-up is using his reconstructed thumb for all his activities including for wearing the lower limb prosthesis. Harvesting the toe from the to be amputated part has the advantages of harvesting more skin, longer length of tendons, vessels, and nerves since there is no need for donor site closure or any concern for donor site morbidity.

## Introduction

Loss of thumb results in functional loss amounting to 40% of hand function. 1 Microvascular toe-to-hand transfer is the gold standard for reconstructing an amputated thumb, typically performed electively. Situations rarely occur where there is an amputation of the lower limb with the same patient requiring thumb reconstruction. We present a case where the great toe was harvested prior to above-knee amputation of a nonsalvageable lower limb and was used to reconstruct the thumb with successful rehabilitation. Increased awareness of the possibility is essential to seize the opportunity of thumb reconstruction sourcing the toe from the limb to be amputated.

## Case Report

**Fig. 1:**
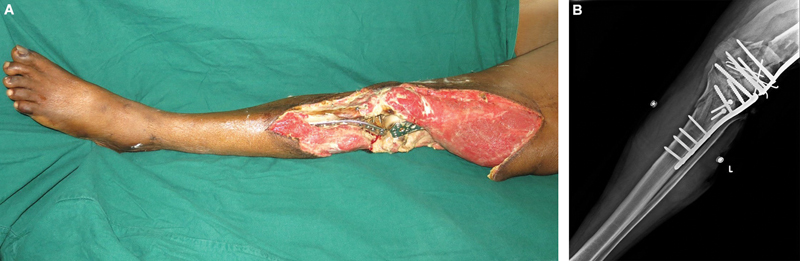
Presenting images of the left lower limb. ( **A** ) Nonsalvageable limb with a Mangled Extremity Severity Score (MESS) of 7 and Ganga Hospital Open Injury Severity Score (GHOISS) of 14. The implants were found to be exposed. ( **B** ) Radiograph showing the severity of the bony injury around the knee making it unstable.

**Fig. 2:**
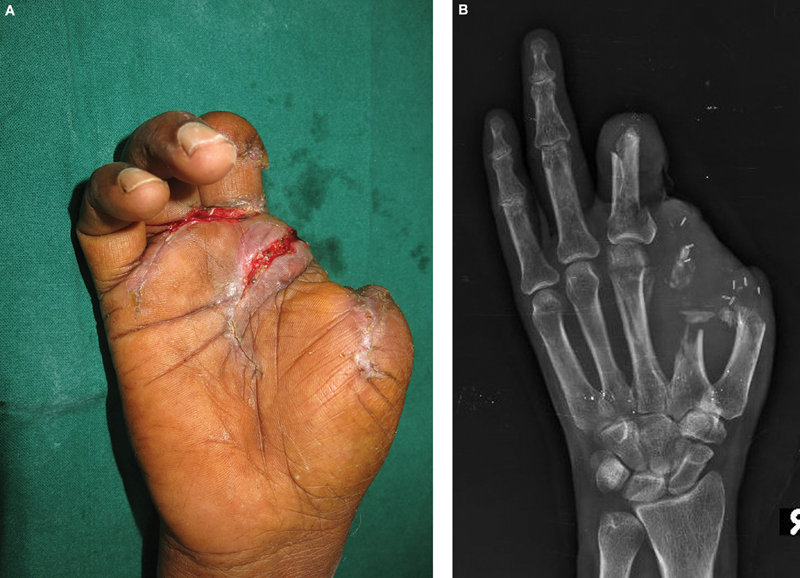
Presenting image and radiograph of the right hand. ( **A** and **B** ) The thumb was amputated at the metacarpal head level. The index and middle fingers were also in an amputated state.

A 59-year-old right-handed man sustained crush injuries to his right hand and left lower limb while attempting to board a moving train. He suffered a grade 3B open supracondylar fracture of the femur and a Schatzker type IV fracture of the tibia with skin loss extending from the middle of the thigh to the middle of the leg. Primary management at another center involved debridement and skeletal stabilization of the left lower limb fractures ( Fig. 1 ). The right hand suffered thumb amputation at the metacarpal head level, index finger through the neck of metacarpal, and the middle finger through proximal phalanx ( Fig. 2 ). He reached our hospital 10 days postinjury for soft tissue reconstruction of the lower limb.

On arrival, we found the lower limb wound infected, an open knee joint with the articular cartilages appearing nonviable. Exposed fracture site revealed desiccated bone. Skin loss extended from the mid-thigh to the middle of the leg anteriorly and near circumferentially at the level of the knee joint ( Fig. 1 ). The knee was unstable. Considering the presence of infected and unstable joint, with possible long-segment bone loss post-debridement, and extensive skin loss, it was decided that the best option would be an above-knee amputation and fitting the stump with a prosthesis. On explaining the time frame of treatment, cost of care, and possible outcome in attempting to salvage versus amputation, the patient chose the option of amputation.

When the patient agreed for amputation of the lower limb, we suggested reconstruction of the amputated thumb with toe transfer to which the patient agreed. The great toe was chosen because the hand had suffered amputation of multiple fingers. We have found that in such instances great toe transfer provides more power to the hand than second toe transfer. 2

**Fig. 3:**
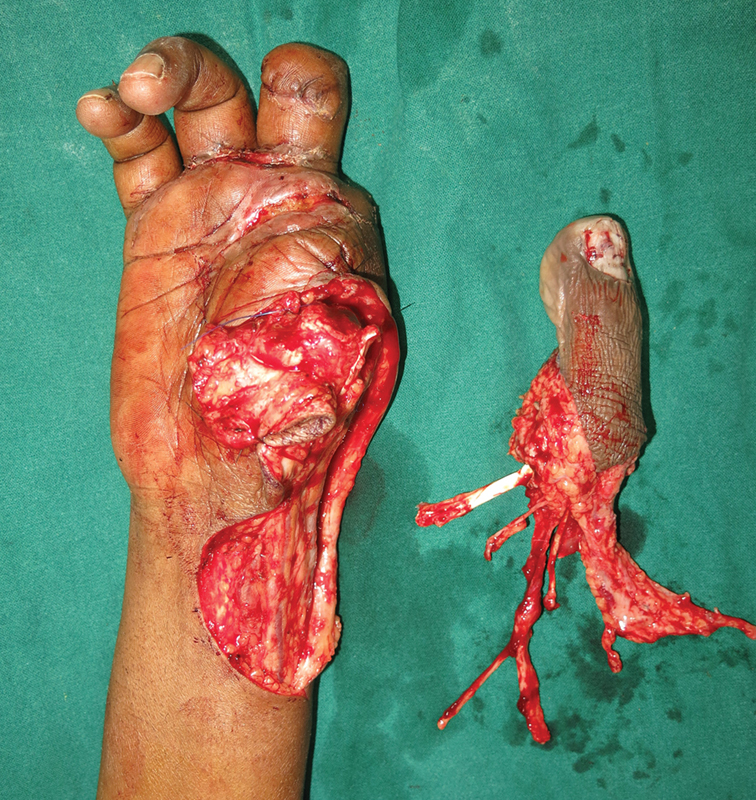
Great toe harvested for thumb reconstruction.

Two teams worked on the patient. One team harvested the great toe and the second team completed the above-knee amputation. The great toe was harvested at the level of the metatarsophalangeal joint, and was fixed to the head of the first metacarpal with a 1.5-mm Kirschner wire. Flexor and extensor tendons and digital nerves were repaired. The dorsalis pedis artery was anastomosed to the radial artery and the saphenous vein was anastomosed to the cephalic vein ( Fig. 3 ). Kirschner wire was removed at 5 weeks followed by therapy. By 6 months, he could pinch and grasp objects, eat and write with his hand, and do all his day-to-day activities.

**Fig. 4:**
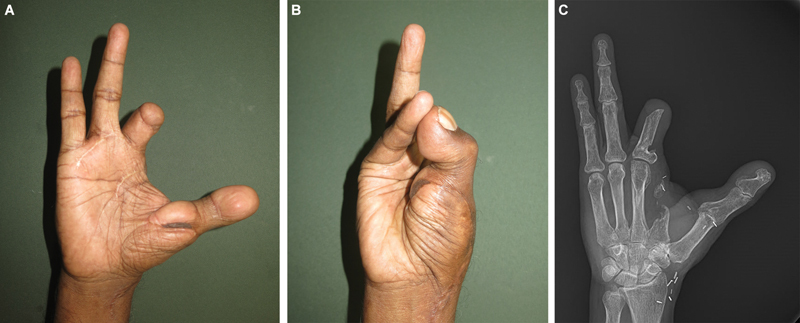
Successful great toe to hand transfer. ( **A** and **B** ) Good position of the new thumb. ( **C** ) Radiograph showing good bone union.

Assessment at 1 year revealed a grip strength of 25% of the normal hand, effective opposition (Kapandji score of 5), and a Disabilities of the Arm, Shoulder and Hand (DASH) score of 37.9. The Jebsen–Taylor score was 77 for the affected hand and 83 for the unaffected hand, which showed that there was no transference of dominant functions to the left hand. The patient was fitted with a prosthesis for walking, and was using the reconstructed thumb for wearing the prosthesis. The pulp-to-pulp pinch strength improved by 25% (2 kg at 1 year) and lateral pinch improved by 40% (2.5 kg at 1 year) ( Fig. 4 ). The Kapandji score improved to 9. Sensations improved from protective at 1 year to diminished light touch on monofilament testing at 5 years. Though the two-point discrimination measured more than 15 mm during the follow-up assessments, the patient was using the new thumb well. The DASH score improved to 31. The patient successfully managed daily activities. Since the hand function is good with the great toe transfer to the thumb, we do not consider a second toe transfer. The patient is satisfied with the functional results and has not opted for any further surgeries.

## Discussion

Toe transfer is the ideal method of thumb reconstruction following amputation. 3 Usually, it is performed electively. Acute primary reconstruction has also been done with proven safety and good outcomes. 4 5 6 7 There are even more advantages in harvesting a toe from the limb to be amputated—more skin can be harvested, longer length of vessels, nerves, and tendons can be taken along without any concern for donor site morbidity. Harvest time is also shorter since there is no need to close the donor site.

In our experience, most patients choose second toe over the great toe for thumb reconstruction due to lesser donor site morbidity. When there is severe mutilation, great toe transfer would provide a better functional result than a second toe transfer with negligible donor site issues, since only parts which otherwise would be discarded are used. 3

Opportunistic spare part surgery has been in the armamentarium of the reconstructive microsurgeon to enhance the quality of life of the individual. 8 9 It exemplifies the 11th principle enunciated by Gillies—“Never throw anything unless you are sure that it is not necessary.” 10 Ability to see the broader picture and making the entire plan rather than for that day, would allow the utilization of tissue “spare parts,” to reconstruct critical defects. This becomes significant since most of these patients would be treated by nonplastic surgeons. The case is presented to revisit this spare part surgery concept and explain the need for referral to higher centers when the possibility is thought of. Reconstruction of the thumb is important in this patient since he has suffered an above-knee amputation and one needs a good hand to help wear the prosthesis.

## Conclusion

The concepts of toe-to-thumb transfer and tissue repurposing in emergencies are well known, but their combination is rarely discussed. Primary care surgeons must be made aware of the possibilities so that timely referral could be made to make “spare part” surgery possible.

## References

[1] Flatt A E Our thumbs Proc Bayl Univ Med Cent 2002 15 04 380 387 16333469 10.1080/08998280.2002.11927870 PMC1276642

[2] Sabapathy S R Venkatramani H Bhardwaj P Mohan M Varadharajan V Toe transfers in mutilated hands: technical considerations to get good outcomes J Hand Surg Glob Online 2024 7 02 362 367 40182874 10.1016/j.jhsg.2024.10.003 PMC11963029

[3] Waljee J F Chung K C Toe-to-hand transfer: evolving indications and relevant outcomes J Hand Surg Am 2013 38 07 1431 1434 23790426 10.1016/j.jhsa.2013.03.020 PMC4192645

[4] Georgescu A V Battiston B Matei I R Emergency toe-to-hand transfer for post-traumatic finger reconstruction: a multicenter case series Injury 2019 50 05 S88 S94 31708087 10.1016/j.injury.2019.10.056

[5] Yim K K Wei F C Lin C H A comparison between primary and secondary toe-to-hand transplantation Plast Reconstr Surg 2004 114 01 107 112 15220577 10.1097/01.prs.0000127803.00139.96

[6] Woo S H Kim J S Seul J H Immediate toe-to-hand transfer in acute hand injuries: overall results, compared with results for elective cases Plast Reconstr Surg 2004 113 03 882 892 15108880 10.1097/01.prs.0000105340.26227.b5

[7] Ornelli M Ruocco G Kaciulyte J Lazzaro L Felici N Immediate vs. delayed toe-to-thumb transfer: is the infection rate greater? Handchir Mikrochir Plast Chir 2019 51 06 434 439 31698486 10.1055/a-0874-2253

[8] Venkatramani H Patel S K Mohan M Muthukumar V Sabapathy S R Emergency foot fillet free flap based on posterior tibial vessels for reconstruction of contralateral heel and sole: a unique spare part surgery J Hand Microsurg 2024 16 01 100004 38854374 10.1055/s-0042-1749443 PMC11127540

[9] Sabapathy S R Venkatramani H Mohan M Zhang D Reconstruction of a monodactylous hand with microsurgical free foot-to-hand transfer in split-hand/split-foot malformation with tibial aplasia Plast Reconstr Surg Glob Open 2020 8 02 e2614 32309073 10.1097/GOX.0000000000002614 PMC7159962

[10] Gillies H Millard D R The Principles and Art of Plastic Surgery 1st ed. Boston Little, Brown and Company 1957 48 54

